# Piecemeal Buildup of the Genetic Code, Ribosomes, and Genomes from Primordial tRNA Building Blocks

**DOI:** 10.3390/life6040043

**Published:** 2016-12-02

**Authors:** Derek Caetano-Anollés, Gustavo Caetano-Anollés

**Affiliations:** 1 Department of Evolutionary Genetics, Max-Planck-Institut für Evolutionsbiologie, 24306 Plön, Germany; caetano@evolbio.mpg.de; 2 Evolutionary Bioinformatics Laboratory, Department of Crop Sciences, University of Illinois at Urbana-Champaign, Urbana, IL 61801, USA

**Keywords:** genome evolution, origin of proteins, ribosome evolution, origin of the genetic code

## Abstract

The origin of biomolecular machinery likely centered around an ancient and central molecule capable of interacting with emergent macromolecular complexity. tRNA is the oldest and most central nucleic acid molecule of the cell. Its co-evolutionary interactions with aminoacyl-tRNA synthetase protein enzymes define the specificities of the genetic code and those with the ribosome their accurate biosynthetic interpretation. Phylogenetic approaches that focus on molecular structure allow reconstruction of evolutionary timelines that describe the history of RNA and protein structural domains. Here we review phylogenomic analyses that reconstruct the early history of the synthetase enzymes and the ribosome, their interactions with RNA, and the inception of amino acid charging and codon specificities in tRNA that are responsible for the genetic code. We also trace the age of domains and tRNA onto ancient tRNA homologies that were recently identified in rRNA. Our findings reveal a timeline of recruitment of tRNA building blocks for the formation of a functional ribosome, which holds both the biocatalytic functions of protein biosynthesis and the ability to store genetic memory in primordial RNA genomic templates.

## 1. Introduction

Uncovering patterns and processes responsible for the origin of life in extant macromolecules is a most challenging proposition. The biological world is largely governed by the functions of protein and nucleic acid molecules. Proteins and RNA make up the molecular machinery of the cell while DNA generally holds its historical repository, its “genetic” memory. The diversity of molecular structures and functions that have been surveyed in proteins and nucleic acids is unprecedented. As of 26 October 2016, 1221 vetted 3-dimensional fold designs defined by one protein classification [1] encompass the structure of 244,326 protein structural domains that hold individually or in combination ~5 million experimental and non-experimental annotations of molecular functions defined by ~9,000 terminal Gene Ontology definitions [2]. Only a relatively small subset of these fold structures are present in each and every organism that has been prospected [3]. Similarly, only 2,474 RNA families have been defined [4], of which only 5 are universal [5]. For decades, molecular biologists have pondered over this diversity as they attempted to explain how life originated in this planet. The genomic revolution has not been forthcoming either. No clear link has been found that explains how the 123,870 models of molecular structure deposited in the entries of the protein data bank (pdb) [6] and their associated functions are encoded in the DNA of the 10,045 genomes and metagenomes that have been completely sequenced (gold database [7]) and that have given rise to 0.55 million uniprotkb/swissprot and ~68 million uniprotkb/trembl protein sequence entries and information on thousands of functional RNA molecules important for probing the workings of the cell. We know there is a code in the memory of life, the genetic code. We do not know how that code maps to the memory of structure and function of proteins, the structural and functional code. Here we argue that this crucial liaison involves transfer RNA (tRNA) and was established very early in evolution once nucleotide cofactors of primordial polypeptides were lengthened into primordial RNA loops. We propose that these nucleic acid loops were capable of interacting stereochemically with evolving protein structure and responding to their molecular makeup. Increases in these interactions canalized both the appearance of genetic memory and building blocks (modules) of RNA with which to construct processive biosynthetic machinery on one hand and genomic memory storage on the other. We review phylogenetic evidence that provide support for these claims and address the properties of the emergent tRNA and rRNA molecular systems viewed fundamentally from the perspective of emerging proteins and genetic information in primordial cells. First, we examine the structures, functions and time of origin (age) of structural domains of proteins defined at the fold family (FF) and fold superfamily (FSF) levels of SCOP, the *Structural Classification of Proteins* [1]. In these studies, the ages of domains are derived from rooted phylogenomic trees built from abundance counts of domains in proteomes [8,9,10]; Second, we use a molecular clock of folds to convert relative age into geological time [10]; Third, the age of tRNA and ribosomal substructures calculated from an exhaustive phylogenomic analysis of thousands of molecules [3,11] is linked to the history of proteins; Finally, we assign ages of helical segments of rRNA to remote tRNA homologies recently identified in rRNA [12], establishing correlations with the ages of corresponding tRNA molecules [3]. The exercise reveals the modular role of tRNA in the early evolution of ribosomes and genomes. The results and implications are remarkable.

## 2. Unity and Diversity in the Evolutionary History of Biological Modules and Systems

**Figure 1:**
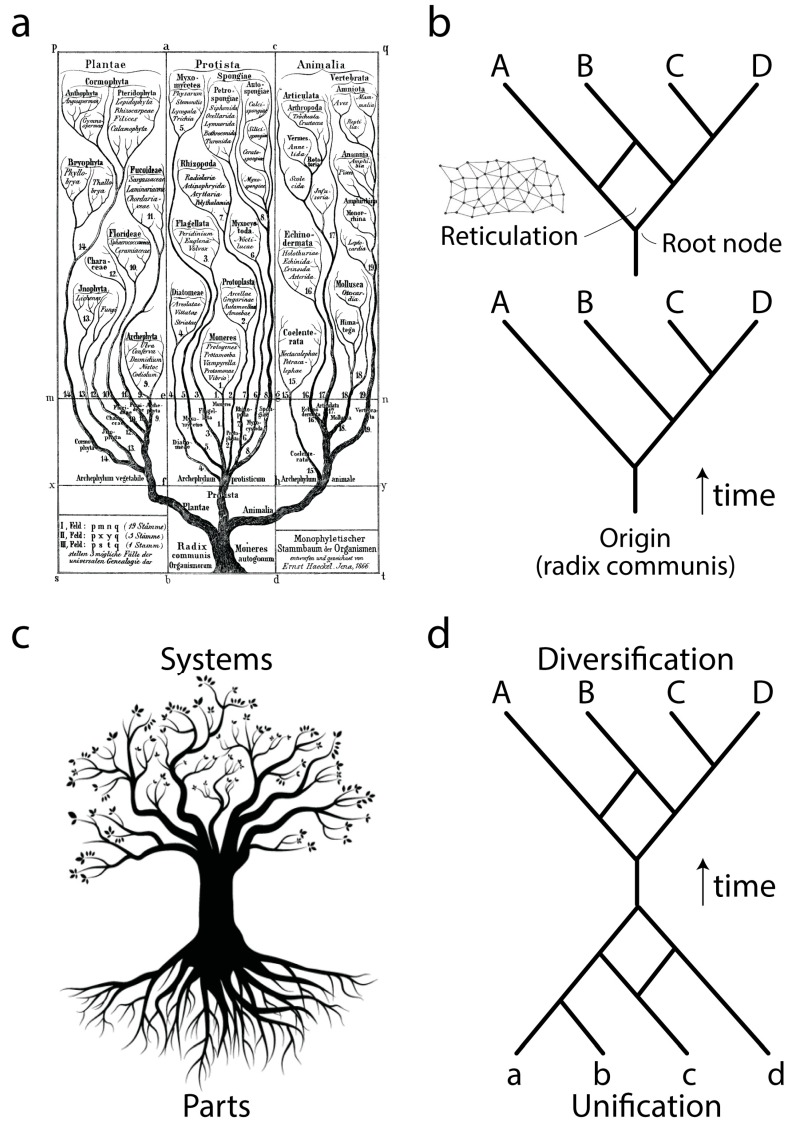
Paradigms governing evolution. (**a**) Tree of life drawn by German zoologist Ernst Haeckel (ca. 1866) depicting the existence of a common ancestor or “*radix communis organismorum*” (the common root of all organisms) unifying diversified cellular life embodied in the leaves of the tree or any transect along its crown; (**b**) In mathematics (graph theory), a tree abstraction can be used to describe the evolution of biological entities, which can be considered either parts of systems or entire wholes. The tree must be rooted to impart a direction and “arrow of time” to its statement of diversification and change. However, tree descriptions can be faulty because multiple evolutionary origins (convergences) are possible when the initial memory of systems is tangled by recruitment or other complicating processes of horizontal exchange. These convergences cause reticulations (see tree with reticulation in top) and in extreme cases “rhizomes” (inset). For example, taxon B has two possible ancestors (one shared with taxon A and the other with taxon C and D), which converge to form its lineage; (**c**) A new paradigm describes the rise of biological parts (modules) from more primordial components and their subsequent diversification. This is illustrated with a tree that shows its trunk separating its root and crown. When considering all biological parts, the tree-like structure describes the evolution of biological systems; (**d**) The abstraction of panel c can be defined by two networks (root and crown networks) joined by a common edge (trunk). This common edge represents the last common ancestor of systems A, B, C and D (members of the crown) as it arises from modular parts a, b c and d (members of the root).

Ever since Darwin evolution has been described using the paradigm of trees (Figure 1a), network abstractions that showcase complex historical processes of diversification (Figure 1b, bottom). The development of cladistics and advanced phylogenetic methodology has shown that biological data exhibits one universal property: vertical traces of genetic memory across time are always complemented with horizontal exchanges of that memory. Thus, the tree paradigm should be considered an oversimplification necessary for the heuristic computational search of optimal phylogenies, hypotheses of history describing the evolution of the biological entities (taxa) that are being studied. Instead, trees with reticulations (sometimes making up reticulated nets or rhizomes; Figure 1b, top) may be more appropriate, especially when studying the evolution of taxa in which processes of horizontal exchange of genetic information override vertical genetic signatures. These scenarios are common in the evolution of bacteria and archaea. Central to evolutionary tree and network thinking is the notion of a common ancestor to the group of evolving entities, a “*radix communis*” that unifies the phylogeny (Figure 1b). This usually takes the form of a “trunk”, a branch leading to a root node exemplifying the hypothetical common ancestor of the entities that are evolving along the branches of the tree or network.

Phylogenetic trees or networks are built from useful biological features of evolving taxa, which are known as phylogenetic “characters”. These characters are usually building blocks (parts) of more complex physical or functional systems (wholes). Molecular examples include amino acids of proteins or nucleotides of nucleic acids. Because parts and wholes are interrelated, trees describing the evolution of systems also describe the evolution of their building blocks (Figure 1c). Under this new paradigm, the evolutionary unification of building blocks results in new emerging systems (defined below), which then diversify. We exemplify this process with a mathematical abstraction (Figure 1d) in which the edges of a primordial root network join to form an ancestor trunk edge. This trunk then diversifies into a crown network of extant entities and their ancestors. Here we focus on the root network of this new abstraction, using structural domains of proteins and central nucleic acid molecules as the subjects of study. We note that this new “hourglass” network paradigm applies to each and every component part of a biological system and that each hourglass does not necessarily occur contemporaneously in evolution. For example, the rise of multidomain proteins from the combination of individual structural domains (reviewed in [3]) was likely preceded by the combination of lower level structural parts to form each protein domain. Here we discuss how this can be made explicit to help us understand processes of macromolecular emergence.

## 3. Memory and the Evolutionary Drivers of Abundance, Recruitment and Accretion

An emerging system in biology must be dynamic and persistent. It must be a natural object with behavior and makeup delimited by a set of interacting component parts (subsystems). Its behavior and makeup must be characterized and individuated from other systems by its cohesion, i.e., by the dynamical stabilities of the component parts when constrained by the system as a whole [13,14]. Persistence refers to the ability of the system to display memory, i.e., to preserve a behavior and make up despite constant perturbation from environments internal and external to the system in question. Within these confines, the emerging system exploits the three fundamental properties of any engineered object, economy, flexibility and robustness [15]. Since these properties are strongly impacted by the way the system perceives both the environment and its internal state, the trade-off solutions that are achieved vary with time and context and have been modeled by a “triangle of persistence” and the system’s environmental history, its “scope” [16]. We note that scope has two components, “umwelt” (the system’s perception of that history) and “gap” (the system’s blind spot, the scope that is not covered by its umwelt). The triangle of persistence was recently used to mathematically explain the existence of a Menzerath-Altmann law of language in the domain makeup of proteins [17]. This law, which states that larger systems hold smaller component parts, manifests by decreasing the length of structural domains when their number increases in multidomain proteins. Thus, the interplay of economy, flexibility and robustness can be made explicit at the biomolecular and biophysical level.

In biology, the memory of a hierarchical biological system (α) increases by increasing the abundance of both its nested parts and wholes. Equation (1) summarizes the process of increasing memory by increasing the number of parts and wholes (that we label *a*) to higher abundance levels (*a’* > *a*, that we label *A*).

(1) $$a\overset{\alpha 1}{\to }A$$

Highly abundant parts and wholes have higher chances of remaining persistent and by doing so enhancing the survival and memory of the system under consideration. For that reason, it is generally unlikely that once high abundance levels *A* are achieved these levels will return to lower levels by loss, unless strong reductive evolutionary forces are at play that are beneficial to the system. This is particularly so when the views of hierarchical systems are global and focus on the higher hierarchical level rather than the local and lower level. Abundance can be increased in many ways but it generally involves the existence of compositional or informational bias. For example, the famous Urey-Miller spark experiments of the 1950’s demonstrate the facile generation of only a limited set of amino acids from the simulated gaseous environments of early Earth [18]. These sets include alanine, glycine, aspartic and glutamic acid, valine, leucine, isoleucine and serine. These same amino acids are overrepresented in salt-induced experimental formation of small dipeptides and polypeptides under prebiotic conditions [19]. Similarly, peptides enriched in alanine, glycine, aspartic acid and valine hold hydrolytic functions and can be produced experimentally by repeated dry-heating cycles and by solid phase peptide synthesis [20]. Finally, these same amino acids are overrepresented in the dipeptide constitution of proteins when globally surveyed in proteomes [21]. Thus, a memory implanted by compositional biases in plausible chemical reactions manifests at different and increasing levels of the hierarchy of life. In our example, they even express in proteins that are encoded by modern genomes. It is particularly noteworthy that this memory has been made mathematically explicit by computer simulations that describe how compositional biases relate to information storage [22].

Memory can also be enhanced by recruitment (also known as cooption or exaptation), the ability to use existent parts in new different contextual environments. Equation (2) summarizes how the process of memory of recruited parts *a* increases when these are recruited by parts *b*.

(2) $$a+b\overset{\alpha 2}{\to }Ab$$

Since recruitment is usually associated with increases of abundance, the abundance of older parts that are coopted by newer ones follow the trends of Equation (1). Examples of recruitment of these kinds are many. In metabolism, a simple analysis of the distribution of protein fold structures in metabolic pathways suggests metabolic networks grow piecemeal and form evolutionary patchworks [23]. More recently, the use of an algorithmic implementation that derives the most plausible ancestry of an enzyme from structural and evolutionary annotations revealed that the recruitment of ancient domain structures by modern enzymes is widespread in metabolic networks [24]. Note that parts need not be physically associated to fulfill Equations (1) and (2) and enable increases of abundance by recruitment. In the case of metabolic networks, the enzymatic parts that are recruited are often loosely associated in the cell but their metabolic functions are established cohesively. Memory can also be enhanced by a related process, accretion. This occurs when parts are recruited pervasively into a system, one at a time, and remain tightly linked with each other. We note that in most cases the recruited parts become physically associated with the growing system, with *b* of Equation (2) representing the growing system and *a* the accreted part. Examples of accretion of these kinds are macromolecular complexes such as the ATP synthases and the ribosome [25,26]. The F-type and A/V-type synthases are multi-subunit complexes responsible for membrane-coupled energy conversion reactions. They produce most of the ATP needed to power cellular processes. Using phylogenomic methods we have shown that the synthase complexes developed gradually by addition of structural domains, starting with the ring structures of the rotating head, followed by the central stalk (the axle), and ending with the structures that regulate their motion (the stators) [25]. Similarly, the ribosome has been shown to grow in evolution by addition of helical segments to the evolving molecules [26], complying with the principle of continuity that sustains evolutionary thinking and explaining the formation of highly sophisticated macromolecular machinery.

Since parts and wholes in biology are highly dynamic entities, only the subset of them that expresses some long-term stability and arise as cohesive element within the expanding hierarchical system will be stable enough to display memory. These parts generally represent *modules*. A module can be defined as a set of integrated parts that cooperate to perform a task [27]. These parts interact more with each other than with other parts of the system, including parts of other modules. Since modules result from the emergent properties of the hierarchical system [15], they must hold history. While defining modules in biology can be challenging, the fact that modules must be “evolutionary units” enables the use of phylogenetic methods to appropriately test definitions of modularity. In this regard and in the absence of clear statements of topographic correspondence, definition of modules through homologies often requires dynamic homology analysis [28] or the use of hidden Markov models [29]. These methods are capable of distinguishing similarity due to common ancestry from similarities due to other causes that are not evolutionary.

## 4. The Usefulness of Abundance in Phylogenomic Analysis

We study the crucial role of abundance by focusing on the evolution of proteins and nucleic acids and reconstructing phylogenetic hypotheses that are grounded in data and computational optimization. Both of these macromolecules are responsible for the rise of biology and genetic memory that we here explore. Methodologically, we take an ideographic (historical and retrodictive) approach that uses information in the protein repertoires of thousands of genomes and advanced tools of phylogenetic analysis to build statements of history, phylogenies of protein parts [3]. The approach takes advantage of the benefits that both molecular structure and abundance provide to the many challenges of phylogenetic reconstruction. These benefits have been discussed in detail elsewhere [30]. Because protein domain structure is several orders of magnitude more resistant to the effects of mutation than its sequence [31], its high conservation levels make structure more suitable for deep phylogenetic exploration. In particular, the slow evolutionary pace of structural change diminishes the chances that the consequences of the “Markov chain convergence theorem” and the “data processing inequality”, which define how time erases useful historical information, limit retrieval of evolutionary history [32]. Furthermore, the use of domain abundance as phylogenetic character avoids the need of alignments in phylogenetic analysis of sequences (the search for similarities of sets of sequences with unknown correspondences but restricted by the lineal order of residues in the sequence). It also offsets the limitations of character independence of phylogenetic reconstruction that plague the use of molecular sequences in phylogenetic analysis; the mere existence of folding and 3-dimensional structure in macromolecules implies character non-independence in a sequence alignment.

The methodology not only builds phylogenetic trees or networks that describe the origin and evolution of protein and nucleic acid repertoires but also tests if the molecular data that is being analyzed holds historical information. This second aspect of the analysis is crucial since it provides experimental support to the link between molecular abundance and history that enables the construction of powerful trees describing the evolutionary history of molecular parts (e.g., the entire world of protein domains or substructures that describe the history of RNA molecules). It also dispels the possibility that molecular structures emerged from lower component parts (e.g., loops, or nucleic acid motifs) by their fortuitous association, driven solely by improvements in functional versatility. Finding phylogenetic signal in the data supports the existence of historical contingency. We note the importance of understanding the meaning of trees of component parts. For example, a “tree of structural domains” holds domains (suitably defined) as taxa (the leaves of the tree) and defines historical relationships (tree topologies) based on domain abundance in the proteomes analyzed (the phylogenetic characters and data analyzed). Since the data matrix makes use of molecular abundance, the tree that is built from the data does not arise from a model of change that involves structural transformations of domains (e.g., [33]). Instead, the historical relationship of different domains is inferred directly from quantitative information in genomic makeup. This “criterion of primary homology” rests exclusively on genomic abundance of individual domains in proteomes, and its validity is permanently tested by mutual optimization of phylogenetic signal in characters and tree reconstruction (an exercise known as Hennigian illumination). Thus, the methodology operates under the Popperian pillars of content of theories and degree of corroboration (see [21] for an explicit elaboration), acknowledging the need of more modern technical definitions of “verisimilitude” for scientific inquiry. The historical signal and reliability of phylogenies of structural domains that have been published are being gradually strengthened by more data (e.g., more proteomes and better sampling of the world of cells and viruses) and better optimization (e.g., improved hidden Markov models and increased background knowledge).

Operationally, the phylogenomic methodology of building trees of parts starts by defining taxa and doing so exhaustively (e.g., all suitably defined structural domains present in proteomes, all substructures of RNA molecules). Properties of this finite taxon set are then studied, such as abundance of structural domains in proteomes or structural or thermodynamic features of RNA substructures in RNA molecules. This generates taxon-character data matrices with data encoded by transformation into alpha-numeric values suitable for optimization with phylogenetic reconstruction software. The software optimizes character changes in all possible unrooted trees (portraying phylogenetic relationships of taxa) during exhaustive or branch-and-bound tree searches or uses heuristic approaches to find optimal solutions according to the maximum parsimony criterion. The most parsimonious trees that are retained are then rooted *a posteriori* using Weston’s generality criterion of derived character states being less widespread in tree branches implemented with the Lundberg method, which reorients and roots the tree by pulling down the branch that yields the minimum increase in character state change. The rooted trees that are recovered are comb-like and can be converted into chronologies of taxa by backward-counting nodes (branch points or bifurcations) from leaves to the root of the optimal trees. This counting defines a relative *node distance* (*nd*) and a scale from *nd* = 0 (most basal and old) to *nd* = 1 (most recent and young), which is used to define how evolutionarily derived is each taxon in the tree of parts. In the case of protein structural domains, we have shown that *nd* correlates strongly with actual time for folds that are linked to markers of the geological record [10]. We use this molecular clock of folds to define the ages of structural domains as a chronology in billions of years (Gy). Given protein-RNA domain interactions that are known, the chronology can be used to transfer age from proteins to interacting RNA by assuming that the age of the RNA molecule is the age of the protein-RNA interaction.

We end by noting that abundance can be coarse-grained into occurrence for phylogenomic analysis, i.e., quantitative valued characters can be reduced to a data matrix of 0 s and 1s, a binary system. While coarse-graining results in some loss of phylogenetic signal, occurrence and abundance generally produce congruent historical statements that can be separately optimized (e.g., [34]).

## 5. The Early Primacy of Peptides, Polypeptides and Proteins in Cellular Environments

“*All language is a set of symbols whose use among its speakers assumes a shared past. How, then, can I translate into words the limitless Aleph, which my floundering memory can scarcely encompass?*”.

—El Aleph and Other Stories, Jorge Luis Borges

In modern biology, peptides and proteins are encoded in genomes and are translated from mRNA into folded polymers that are functional. Similarly, transcribed non-coding RNA folds into functional forms. The 3-dimensional molecular structures of these macromolecules tend to become compact when they collapse into stable conformations and abandon the benefits of interacting with the aqueous environment that forces them to maintain the unfolded state. The folded structures provide a fundamental scaffold to constrain in favorable conformation the small subset of amino acid residues responsible for protein functions. These residues are generally lodged in pockets on the surface of the protein, though networks of residues throughout the molecule play also roles in allosteric regulation and protein stability. The small subset of residues is mostly associated with the unstructured regions of proteins, suggesting that the complex arrangement of secondary structures (helix, strand and turn) collapses into 3-dimensional topologies that best respond to the needs of the more dynamic and functional regions of the protein. In fact, phylogenomic analysis has shown that folding speed and flexibility are beneficial traits that are fostered in evolution [35] and that flexible loop regions were enriched in proteins by the rise of genetics [21]. Moreover, structural flexibility is even a conserved feature in the assembly of protein complexes [36]. Thus, protein flexibility appears a crucial property in protein evolution at different levels of the hierarchical molecular system, even in the absence of a primordial biology that could translate nucleic acid information into proteins.

Structural domains in proteins are considered evolutionary units. Any statement about their history that is obtained using phylogenetic approaches or hidden Markov model libraries (e.g., timelines of the evolutionary appearance of domains, trees or networks of domains, domain groupings) will relate solely to their history and not to the history of other modules and evolutionary units that exist at lower or higher level of the hierarchical molecular system. During the past decade we have generated phylogenomic reconstructions of the evolution of structural domains at the different structural abstractions of SCOP: folds, FSFs and FFs, from deeper to shallower evolutionary views of molecular structure (reviewed in [3]). The emergent picture of molecular evolution derived from domain history is largely congruent regardless of the level of abstraction [9] or the classification system that is used to define structural domains [37]. The very early and oldest domains are fully dependent on cellular membranes. Thus, the first proteins appear to have emerged enclosed in primordial containers (cells) and evolved from there to form the wide diversity of globular proteins that currently exist. A devil’s advocate however could challenge any inference derived from annotations of historical timelines by claiming bioinformatic associations of functions and structures say nothing about early historical processes. Indeed, one must assume that modern definitions of functions and structures can be used to interpret those that existed in the past. In other words, we must consider that viewing past events with a modern “lens” is a valid approach. This may not always hold [32] and more philosophical, mathematical and biological elaborations of the implications of considering modern entities as relics of the past await development. The early rise of cellular containers as single or multi-layer vesicles is however supported by the existence of amphiphilic molecules in meteorites that organize themselves spontaneously into liposomes in the laboratory and the possible important role of meteoritic influx on the environments of early Earth (see discussions in [9,25]). Similarly, plausible prebiotic synthesis of membrane constituents exist that could explain their early and abundant formation, notably aided by the effects of clays and other mineral deposits. Finally, structural canalization can be invoked as important force that freezes in time the structure of molecules making these structures highly conserved at evolutionary level [38]. While the activity of this force throughout all levels of structural complexity remains to be explored, canalization appears an important and general principle of conservation in biology that shields the effects of the environment on the organism and may represent an inevitable consequence of complex processes [39].

**Figure 2:**
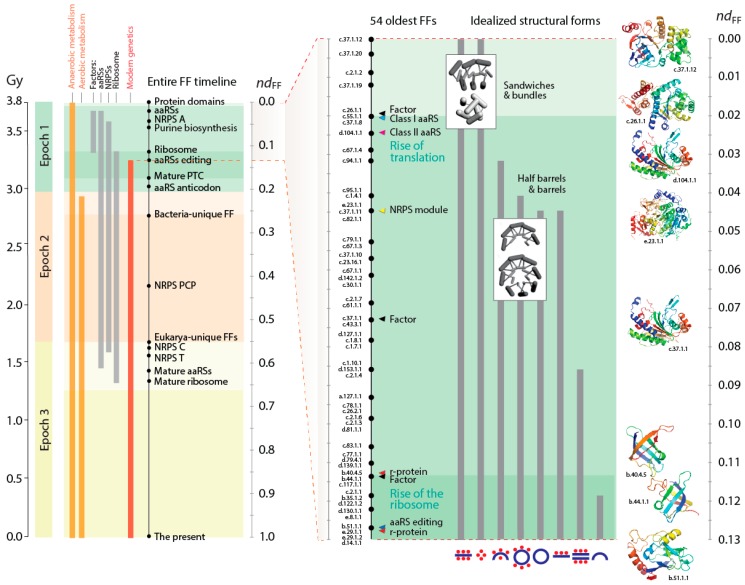
Timeline describing the evolution of structural domains responsible for the primordial components of metabolism and translation. The timeline was derived directly from the tree of FFs reconstructed from free-living organisms. Ages are given as node distances (*nd*_FF_) and geological time in billions of years (Gy). Time flows from top to bottom. The three evolutionary epochs of the protein world, “architectural diversification” (epoch 1), “superkingdom specification” (epoch 2), and “organismal diversification” (epoch 3) (see definition in [8,9]) are indicated with different color shades. Fundamental structural and functional discoveries are identified with circles along the timeline. The inset describes in detail the evolutionary timeline of the 54 most ancient FFs, showing examples of 3-dimensional models and idealized structures with diagrams representing helices with red dots and sheets of strands with blue lines. Colored arrowheads indicate FFs associated with the listed functional discoveries. aaRS, aminoacyl-tRNA synthetase; NRPS, non-ribosomal peptide synthetase; PTC, peptidyl transferase center; ;PCP, peptidyl carrier protein.

A previous study mapped in detail the first evolutionary appearance of the oldest 54 FFs and traced a number of properties of these protein structures, including their ability to bind cofactors, interact with RNA, and display broad molecular movements and flexibility [9]. The set was selected because it laid the foundations for both the metabolic and translation machineries [8,9]. The very early timeline of FFs, which is described in Figure 2, showed that the first four FFs were the ABC transporter ATPase domain-like family (c.37.1.12), the extended and tandem AAA-ATPase domain families (c.37.1.20 and c.37.1.19) and the tyrosine-dependent oxidoreductase domain family (c.2.1.2), all of which exist in highly structured cellular environments. The functions of these FFs are linked to the start of modern metabolic networks, providing hydrolase and transferase functions needed for nucleotide interconversion, storage and phosphate transfer-mediated recycling of chemical energy, and terminal production of beneficial cofactors (e.g., [40]). The early evolution of metabolism in association with nucleotide cofactors culminated with the appearance of the first enzymes of the biosynthetic pathways of nucleotide metabolism 3.5 Gy-ago and the completion of a functional biosynthetic pathway ~3 Gy-ago, which coincides with the rise of a functional ribosome [41]. These coordinated developments suggest the coevolutionary need of a steady supply of nucleotide precursors internal to the cell for the synthesis of large RNA molecules, large enough to store genomic information and fulfill the ribosomal role of processive biosynthesis. The rise of aerobic metabolism at about that time (~2.9 Gy-ago) ultimately results in the great oxygenation event (GOE) of our planet that occurred 2.45 Gy-ago [42], which coincides with the rise of superkingdom-specific domain structures and early organismal diversification that we call the epoch of “superkingdom specification” (Epoch 2; Figure 2).

**Figure 3:**
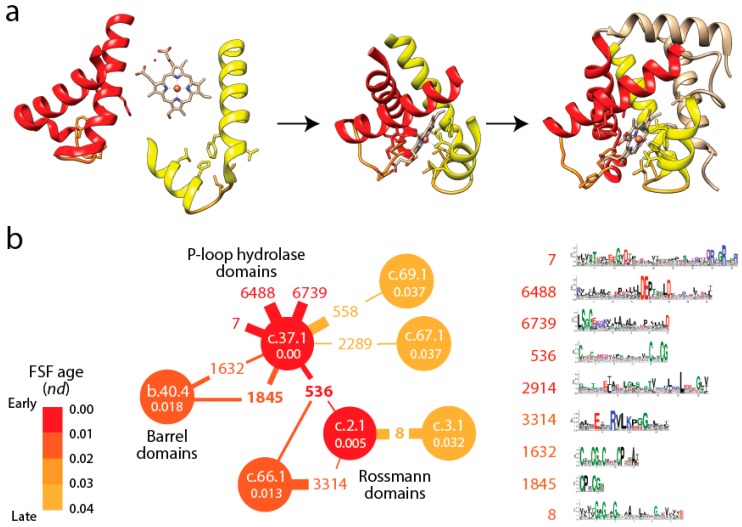
The evolutionary rise of structural domains from supersecondary structural motifs. (**a**) An illustration of the process with the haemoglobin molecule (PDB entry 1THB). Two main elementary polypeptide loops (colored red and yellow) with αα-hairpin structures have sites capable of binding to a protoporphyrin IX-iron complex (heme) for oxygen transport when they come together in space. The evolutionary joining of these loop structures into a single molecule and further growth by addition of extra α-helices produces the modern structural domain of the α-chain haemoglobin; (**b**) Linking evolution of the oldest and most abundant FSF domains (circles) and EFL motifs (numbers) using a bipartite graph. The graph describes the most connected subnetwork of a bipartite network that describes how FSF share EFLs in proteins [48]. This subnetwork is also the oldest. The age of FSFs (*nd*) is indicated inside circles. The P-loop hydrolase fold (c.37.1, *nd* = 0) is the most connected FSF and EFL 536 and EFL 1845 the most connected loops in the subgraph and in the entire network that is not shown. Prototype logos in the right show amino acid residue frequencies in sequence sites of the most ancient EFLs and a clear pattern of EFL length decrease with age that extends to the rest of the graph. Edges represent EFL matches to domains; their width is proportional to the number of matches. FSFs are labeled with SCOP *concise classification strings* and EFLs with prototype numbers*.*

The P-loop containing nucleotide triphosphate (NTP) hydrolase fold (c.37) is the first folded structure of the timeline of FFs (Figure 2). It appears for the first time associated with a primordial bundle, the predominant structure of proteins associated with membranes. The c.37 structure holds a “Rossmann-like” α/β/α-layered design that “sandwich” a sheet of strands between helical segments. This layered design dominates the topologies of many subsequent basal FF structures, including 36 of the 54 oldest FFs. The primordial appearance of this fold confirms once again abundant evidence from phylogenetic reconstruction suggesting that the layered structure was responsible for the first compact protein modules (beginning with [43]). The primordial α/β/α-layered structure has special properties related to lower level structural organization that are very relevant. The structural and functional diversity of proteins can be described by a combinatorial interplay of “supersecondary” structures, modular-like arrangement of helix, strand and turn segments (e.g., αα-hairpins, ββ-hairpins, βαβ-elements), that act as lower level evolutionary building blocks of protein folds and biochemical diversity [44,45]. These supersecondary motifs are generally ~25–30 amino acid residues long and in most cases form recurrent loop structures, many of which determine biochemical diversity [46] and protein flexibility [21]. In evolution, these so called “elementary functional loops” (EFLs) likely combine with each other to better bind cofactors and exert molecular functions (Figure 3a). In fact, the history of these EFLs can be traced back to a small set of loop prototypes, which represent collectives of many sequences embedded in proteins and capable of collapsing into stable loop structures. These EFLs are likely stabilized by the formation of van der Waals locks [47]. An analysis of the most abundant of these EFL prototypes revealed they were associated with a small set of folds defined at the FSF level of the SCOP hierarchy [48].

Figure 3b shows a small subnetwork of the most abundant EFL prototypes and their FSFs, with the ages of FSF mapped onto the network and transferred to EFLs [49]. The bipartite network showed that the P-loop hydrolase FSF (c.37.1) was the most connected hub, benefiting from the assembly of numerous EFLs. In particular, the EFL 536 hub links c.37.1 to the NAD(P)-binding Rossmann fold FSF (c.2.1) that holds the ancient tyrosine-dependent oxidoreductase domain FF. Remarkably, the small subnetwork contains the oldest FSFs of the EFL-FSF network, supporting the fundamental evolutionary link of abundance and time of origin of modules and the special properties of the α/β/α-layered design for building domains. The fact that the history of domains matches inferences from the bipartite network suggests domains structures assembled from loops to form larger and more stable folded structures and that loop ligations were more prone to form stable folded structures (given biases of prebiotic amino acid constituents) in areas of sequence space that materialized into the c.37 fold.

This primordial α/β/α-layered design of the most ancient FFs has also special properties related to amino acid usage. The c.2.1.2 structure for example uses almost exclusively amino acids encoded by the GC-rich half of the codon table and its genes have multiple open reading frames [50]. This appears to indicate that these enzymes acquired their fold structures earlier than a diversified genetic code. Similarly, the dipeptide make-up of protein domains appearing early in the timeline is enriched in hydrophobic amino acids and underrepresented in dipeptides participating in flexible loop regions, suggesting protein flexibility was an important driver for the rise of genetics [9,21]. Enrichment patterns suggest hydrophobicity of dipeptide make-up of the first FFs and a primordial association with membranes. Their rigid protein structures lacking flexible arms and showing limited motions is compatible with standard enzymatic functions. We note however that mutation saturation of sequences has probably replaced the amino acid repertoires present in ancient domain structures with amino acids of the modern 20+ repertoire and that FF structural cores have been decorated with additional structures of much more recent origin, probably harboring all possible amino acid sites. Thus, more modern processes of change complicate inferences derived from sequence analysis.

## 6. The Late Appearance of Interactions with RNA

A number of FFs appeared ~3.7–3.6 Gy-ago (*nd*_FF_ = 0.02–0.045) after the rise of metabolism (Figure 2). These structures catalyzed crucial acylation and condensation reactions involved in aminoacylation of tRNA bound to aminoacyl-tRNA synthetases (aaRSs) or phosphopantetheinyl arms of carrier proteins that are part of non-ribosomal peptide synthetase (NRPS) complexes. These structures, which made their debut before ribosomal proteins in the timeline of FFs, are also part of the catalytic makeup of enzymes important for fatty acid biosynthesis. The first four FFs of this group involve class I aaRS catalytic domain (c.26.1.1), class II aaRS and biotin synthetases (d.104.1.1), G proteins (c.37.1.8) and actin-like ATPase domain (c.55.1.1) FFs. All of them have the α/β/α-layered Rossmannoid design and three of them define the catalytic domains of aaRSs and structures of elongation factors that are central for translation and the specificity of the genetic code. Translation therefore appears to have metabolic origins that predate the appearance of the ribosome [8]. We note the profound implications of the phylogenomic timeline, especially for proponents of the ancient “RNA world” theory that dominates current thinking in origin of life research. While we have discussed the feasibility of this theory elsewhere [25], we ask the reader to keep an open mind when considering the alternatives suggested by phylogenetic evidence that will follow.

Before discussing further implications of the timeline, we want to emphasize the putative environment that fostered all of these structural innovations. Without a genetic memory, the systems had to rely exclusively on biases of the emerging polymers, the prebiotic and biotic chemistries surrounding their functions, and the physical constraints imposed by the emerging cellular systems. The sequence and structures of proteins that we study today have been the subject of up to ~3.8 Gy of continued optimization, of course within the constraints of structural canalization. During the first few hundred million years, those same macromolecules could have not achieved the levels of compactness of modern folds nor the functional efficiency and specificity of the modern macromolecules for several reasons. Evolutionary optimization through mutational change (read compositional variation) could have not covered enough sequence space and any diffusion by random walk had to be faulty and limited by frequent loss and absence of strong selective constraint. We therefore envision that molecules spent considerable time in conformations that were unproductive but were still able to advance optimization through the compositional codes that were slowly materializing. This probably involved favoring limited sets of building blocks, smaller molecules, and smaller patches of inter-molecular interactions. It is highly likely that a multitude of reactants and chemical reactions was available for probing in billions of combinations throughout the entire planet and that only those fortuitous successes would have spread to the rest of the cellular systems through rather free cellular exchange. This necessitates “porous” membranes and smaller molecules than those of today’s biology. For example, it would be non-productive to combine emerging domains into larger ensembles during that time. Indeed, phylogenetic analysis suggests that it took and additional ~2 Gy to fully develop the benefits of domain combinations in multidomain proteins [51].

**Figure 4:**
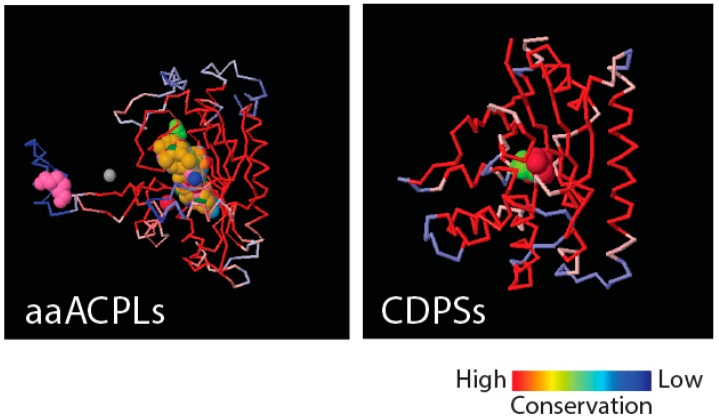
Structural relics of ancient aaRS ligases and dipeptidases. A structural alignment of aaACPL B110957 (PDB entry 3PZC) to the d.104.1.1 FF catalytic core of class II SerRS enzyme from a metanogenic archaeon (entry 2CJ9), which is its closest structural neighbor (Z = 26.8; RMSD = 2.7 Å), is shown in the left. A structural alignment of CDPS AlbC (entry 3OQV) to its best match, the c.26.1 FF catalytic core of a class Ic TyrRS enzyme from an archaeon (Z = 10.0; RMSD = 3.2 Å) is shown in the right. A color scale shows structural alignment conservation used in the tracing of polypeptide backbones. The proximity in DALI structural neighborhoods of aaACPLs and CDPSs to aaRS catalytic cores with major groove specificities suggests a deep evolutionary link to archaic founders of aaRS biosynthetic activities [21].

Implicit in the evolutionary appearance of tRNA-associated FFs is the development of stereochemical interactions between molecules that could jumpstart both “translation specificity” and “genetic memory”. We have proposed a model of emergence of genetics in which molecular interactions define: (i) specificities of an emerging genetic code in “identity elements” of the nucleic acid molecule; and (ii) corresponding FF enzymatic activities responsible for tRNA aminoacylation and the formation of peptide bonds [21]. A corollary that follows from this model is that stereochemical interactions were established between small polypeptide and nucleic acid molecules that were already “structured” by molecular folding. This implies that the FF structural cores had already assembled from small EFLs by statistically biased condensation reactions and were developing archaic aminoacylation and ligation activities for cellular persistence. We stress that the dual role of stereochemical interactions is needed to explain the hidden evolutionary link between the specificity of tRNA identity elements and information in the structure of proteins, and at the same time, explain the selective forces that could be at play. We posit that the hidden link is the formation of dipeptide molecules from pairs of aminoacylated tRNAs by primordial aaRS urzymes [21]. Figure 4 shows that class II aaRS and biotin synthetases (d.104.1.1) and class I aaRS catalytic (c.26.1.1) domains responsible for SerRS and TyrRS aminoacylation activities, respectively, have close structural homologues in amino acid-[acyl carrier protein]-ligases (aaACPLs) and cyclodipeptide synthases (CDPSs), respectively. Note that aaACPLs are relatives of NRPSs and CDPSs are dipeptidases that produce dipeptides from sets of two aminoacylated tRNA. These strong structural homologies are evolutionarily deep. They reveal highly conserved structural protein cores that are putative founders of archaic biosynthetic activities needed to jumpstart primordial genetic and structural codes. To test if indeed dipeptidases were involved in providing building blocks for the structuring of protein domains, we looked for biases in the dipeptide make-up of FFs appearing prior to anticodon binding domains, and found significant biases (*p* < 0.05) against flexible loop regions but favoring turns and bends in the initial FFs [21]. This suggests that genetics arose from biases in the 400+ word vocabulary of dipeptides that makes up proteins and a transition from rigid to flexible protein structural cores.

## 7. Defining a Natural History of Protein Catalytic Mechanisms and Their Interaction with Cofactors

**Figure 5:**
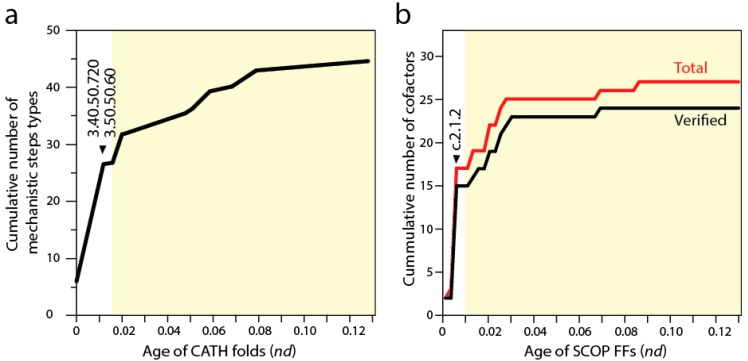
The very early accumulation of new mechanistic steps (**a**); and cofactors (**b**) in evolutionary timelines of structural domains. Mechanistic step types were taken from annotations in macie [52]. Relationships that exist between cofactors and FFs were derived from the procognate and the PDB databases [9]. The total cofactor dataset contains both experimentally verified cofactor-structure relationships and relationships that are not. The most ancient CATH homologous superfamilies and SCOP FFs were arranged by their age (*nd* values). White shaded areas involve domain structures that do not interact with RNA.

Biocatalytic mechanisms are chemical transformations of organic compounds facilitated by protein enzymes and other natural catalysts. In turn, cofactors are “helper” non-protein chemical compounds required for biomolecular activity. Mechanisms and cofactors must reside in special pockets of the enzymatic structure (e.g., active sites) for them to be effective. Recent studies traced the appearance of biocatalytic mechanisms and associated cofactors in structural domain evolution [9,52]. Phylogenomic trees reconstructed from a structural census at the “homology superfamily” level of the CATH classification system (analogous to the FF level of SCOP) allowed to trace the mechanistic step types of the fold structures [52]. Each mechanistic step type is one of 51 mechanistic annotations in the macie database that are used to describe the chemistries underlying enzymatic activities. The basal P-loop containing NTP hydrolase fold (3.40.50.300) introduced the mechanistic steps that are most widely spread in enzymes, including “proton transfer”, “bimolecular nucleophylic addition”, “bimolecular nucleophylic substitution” and “unimolecular elimination by the molecular base”. However, it was two of the following three CATH structures that added almost half of all 51 mechanistic annotations, the NAD(P)-binding Rossmann-like domain (3.40.50.720) and FAD/NAD(P)-binding domain (3.50.50.60) (Figure 5a). These structures preceded the inception of domains that interact with RNA, the Hups (3.40.50.620) α/β-layered domains of aaRSs, which introduce the single mechanistic step of “intramolecular elimination” needed to fulfill their aminoacylation reactions.

A similar progression can be seen by studying the use of cofactors by SCOP FFs in the timelines [9] (see Figure 2). The first appearance of domain interactions with cofactors inferred by cofactor annotations in entries of the procognate and pdb databases revealed the primordial use of ATP and ADP by the c.37.1 structure (Figure 5b), an observation previously intimated from the distribution patterns of small molecule ligands in proteins [53]. However, the Rossmann fold of the c.2.1.2 FF that followed added almost half of all known cofactors of proteins. This burst matches the substantial rise of mechanistic steps immediately preceding the appearance of catalytic domains of aaRSs and protein interactions with tRNA. The finding supports the long held idea of RNA originating from ligation of precursors that were acting as cofactors (e.g., [54]). However, and in contrast with many RNA world-inspired proposals, these ribotide cofactors were being synthesized in pockets of the primordial α/β/α-layered structures. This is compatible with the observation that aaRSs are able to form a wide variety of dinucleoside oligophosphates in the presence of amino acids (e.g., [55]), a property that is also shared by NRPS domains. We therefore hypothesize that the α/β/α-layered structures fostered nucleotide ligations that extended suitable combinations of nucleotides to form longer polymers and that this interplay naturally materialized in rudiments of the genetic code within the confines of an increasingly more complex ribonucleoprotein molecular world. Conversely and in parallel, the α/β/α-layered structures could have also facilitated the ligation of dipeptide and small peptides to form larger molecules. As mentioned above, the structures of catalytic domains of aaRSs can form dipeptides with the aid of tRNA molecules (e.g., [56]), a molecular function that left relics in the dipeptide makeup of proteins [21].

## 8. The Coevolutionary History of Emerging tRNA, rRNA and Proteins and the Rise of Genetics

**Figure 6:**
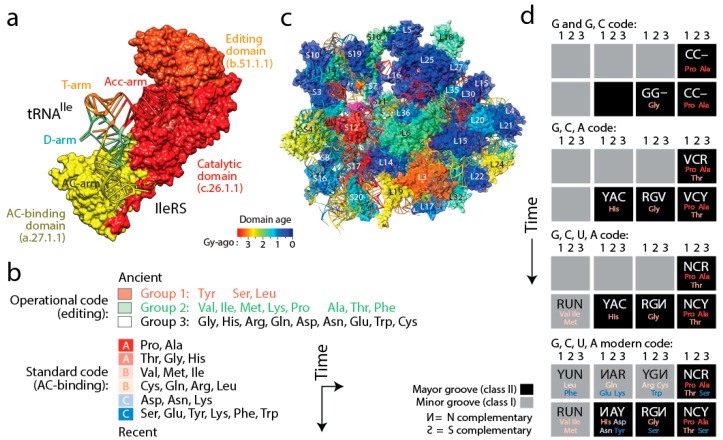
Coevolution of proteins and nucleic acids to form the evolutionary cores of translation machinery and genetics. (**a**) The age of the domains of aaRSs, exemplified by IleRS (PDB entry 1qu2), match the age of the interacting arms of their tRNA isoacceptors. The oldest acceptor (Acc) arm interacts with the oldest catalytic domain and the more recent anticodon (AC) arm interacts with the more recent AC-binding domain [21]; (**b**) Two codon systems evolved sequentially but acted redundantly, one delimiting amino acid charging and the other codon specificity. Phylogenomic analysis dissects their history [21]; (**c**) The ribosomal complex with ages of ribosomal proteins and rRNA helices traced on an *Escherichia coli* structural model of the ribosomal core [11]. Note the very ancient and central translocation core of helix 44 and ribosomal proteins S12 and S17; (**d**) Alphabet evolution of the “standard” genetic code [21]. Ancestries of tRNA-aaRS binding were mapped most parsimoniously onto the condensed Rodin & Rodin’s *vis-a-vis* degenerate genetic code representation, taking into consideration anticodon loop identity elements. This timeline of late genetic code expansion was indexed with major and minor groove modes of tRNA recognition in the aaRS enzymes. N = G, C, U, A; V = G, C, A; R = G, A; Y = C, U; S = G, C.

If indeed emerging domains interacted with initial tRNA cofactors, then it is possible to envision that aaRS enzymes coevolved with tRNA during the rise of genetic code specificities and that tRNA coevolved with the emerging ribonucleoprotein structure of the ribosomes. Coevolution is here defined as the coordinated succession of structural changes mutually induced by the increasingly interacting and growing protein and nucleic acid molecules in their quest to fold into more stable and functionally efficient structures that would provide enhanced stability to primordial cells. Using phylogenomic reconstruction we have been able to support both of these coevolutionary assertions with considerable data. Phylogenetic analysis of thousands of RNA molecules and millions of protein structural domains allowed reconstruction of phylogenies and evolutionary timelines of the history of tRNA amino acid charging and anticodon-binding specificities of tRNA [20] and the history of ribosomal accretion [11]. The relative ages of structures of aaRS domains, ribosomal proteins, tRNA and rRNA drawn directly from the phylogenetic trees were indexed with structural, functional and molecular contact information and mapped (by color) onto three-dimensional models of individual molecules and ribosomal complexes (Figure 6). Four important coevolutionary patterns were revealed.

**Figure 7:**
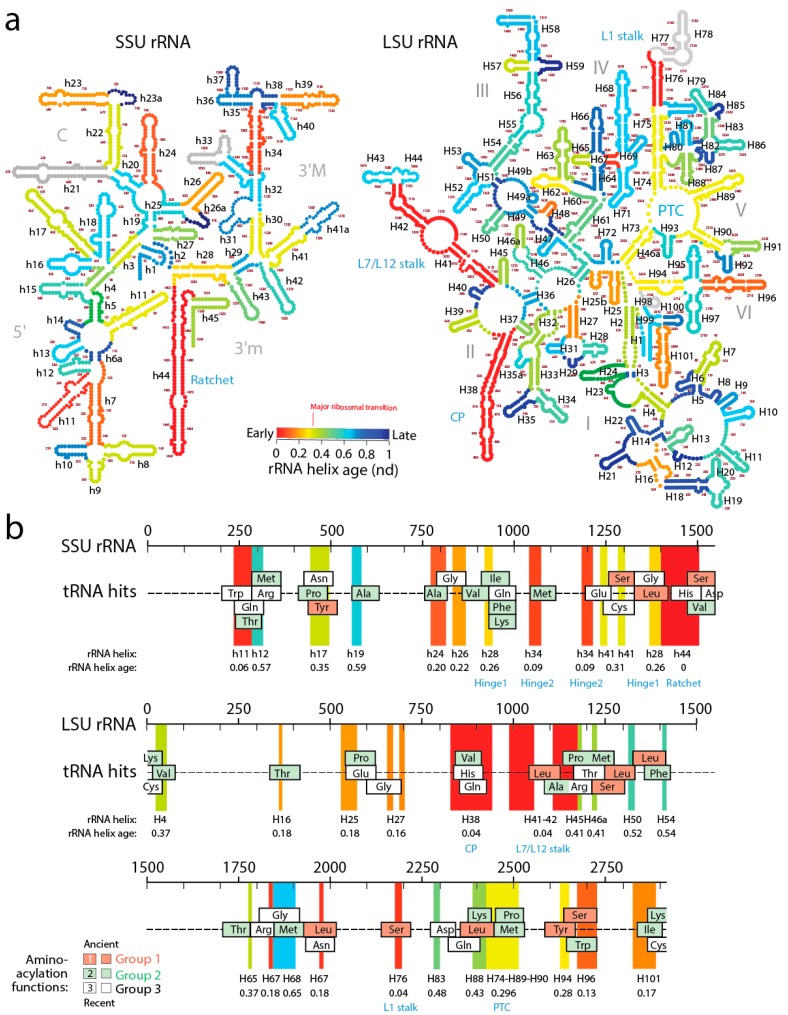
Revealing the gradual formation of a functional ribosome by accretion of tRNA building blocks. (**a**) Secondary structure models of the small (SSU) and large (LSU) subunits of ribosomal rRNA from *Escherichia coli* with helical segments colored according to their relative age (*nd*); (**b**) Mapping of tRNA homologies onto rRNA sequences and tracings of the projection of the oldest helical segments of rRNA that encompass the tRNA homology hits, colored according to age. tRNA homologies as indicated with squares colored according groups of aminoacylation function, with Groups 1 and 2 holding editing functions.

(i) The history of aaRS catalytic, editing and anticodon-binding domains matched the history of tRNA charging and encoding [21] (Figure 6a). These coevolutionary patterns allowed to infer a history of progression of specificities for both the “operational” genetic code of the acceptor arm of tRNA and the “standard” genetic code of the more derived anticodon-binding stem of tRNA (Figure 6b). Since specificity determinants in tRNA result from interaction with the synthetases, the progression describes the rise of the aminoacylation specificities of tRNA isoacceptors. The first specificities involved pre-transfer and post-transfer editing and trans-editing activities responsible of sieving amino acids by size in the active sites of the catalytic domains of the synthetases. These specificities involved 11 of the 20 standard amino acids, which were split into two groups. Group 1 specificities were associated with the older type II tRNA structures holding a variable arm. Group 2 were associated with standard type I tRNA cloverleaf structures. These interactions involved the acceptor stem of the tRNA molecule, the oldest of the molecule [57]. They delimited the operational genetic code, probably in absence of a fully functional ribosome and a full cloverleaf structure. In turn, codon specificities were determined by specific anticodon binding domains in interaction with the more modern anticodon stem of tRNA and appeared much later in the timeline, ~3 Gy-ago. The development of this more modern “standard” genetic code produced its own timeline of codon specificities (Figure 6b). Thus, protein history unfolded separate timelines of amino acid charging and codon recognition, which we had already intimated in an earlier study [58], and revealed coevolution of the emerging domains and nucleic acid cofactors.

(ii) A similar analysis of the evolution of the structure of rRNA and ribosomal proteins of the small (SSU) and large (LSU) subunits of the ribosome produced an evolutionary timeline of accretion of the universally conserved ribosomal complex [11] (Figure 6c). The age of rRNA helical regions (see Figure 7a) and interacting domains of ribosomal proteins coevolved to form a fully functional ribosomal core. The oldest protein (S12, S17, S9, L3) appeared together with the oldest rRNA substructures responsible for decoding and ribosomal dynamics 3.3–3.4 Gy-ago. These structures include the ratchet and two hinges of SSU rRNA and the L1 and L7/L12 stalks of LSU rRNA important for ribosomal movement of tRNA in the complex. While protein-RNA coevolution manifested throughout the timeline, the appearance of RNA substructures at first occurred in orderly fashion until the formation of a 10-way LSU and 5-way SSU junctions, at which point a “major transition” in ribosomal evolution occurred 2.8–3.1 Gy-ago. This transition brought ribosomal subunits together through inter-subunit bridge contacts. It also stabilized loosely evolving ribosomal components and developed tRNA-interacting structures and a fully-fledged peptidyl transferase center (PTC) with exit pore capable of protein biosynthesis. Thus, ribosomal history also showed gradual coevolution between RNA and proteins.

(iii) Coupling the evolutionary timelines of tRNA and rRNA structure with annotations of their interactions with protein domains revealed that the tRNA cloverleaf structure was already fully formed when the PTC appeared in evolution [59]. This was previously intimated directly from phylogenetic analysis of ribosomal history [11]. Thus, fully formed tRNA molecules played other roles before being recruited for processive protein biosynthesis, perhaps as cofactors of peptide-producing dipeptidases and ligases. A more detailed elaboration of our data-driven hypothesis for the origin of translation and genetics can be found elsewhere [9,21].

(iv) Finally, tracing ancestries of tRNA-aaRS binding in a condensed code representation of primordial complementarity indexed with major/minor groove modes of tRNA recognition revealed gradual evolution of the genetic code (Figure 6d). Mappings showed the early use of major groove recognition and the second and first codon positions. The early codes were associated with small and hydrophobic amino acids. The coding of Pro, the founder, was based only on C and already used second and first code positions (identity elements G35 and G36). The code soon expanded into a duplex code by adding G to its alphabet. The use of a third codon position (G34) for the first time with Thr and then His (the last two initial recruitments of the c.51.1.1 FF) expanded the alphabet to a triplex code that used C, G and A. Finally, the “yin-yang” complementarity pattern of the condensed code representation was finally fulfilled with the last recruitment of the a.27.1.1 FF once the modern tetraplex code was in place.

The existence of two codes embedded in the acceptor stem and in the anticodon stem of tRNA has recently received additional support from a study that shows that the acceptor and anticodon stem determinants code for size and polarity of amino acid residues, respectively [60]. This matches the differential encoding of information in the top and bottom half of the tRNA molecule and the role of editing and anticodon binding recognition that differentiate these two sequential and apparently redundant codes [21].

## 9. Accretion of tRNA Building Blocks Forms Functional Ribosomes

A recent study generated lists of non-overlapping alignments between tRNA and rRNA molecules using a pairwise global alignment method implemented with the LALIGN algorithm without end gap penalties and using default parameters [12]. The study uncovered a number of remote homology hits, often overlapping, which suggested both subunits of the ribosome were built piecemeal from primordial tRNA molecules (Figure 7). The finding is significant as it supports the hypothesis anticipated by David Bloch and his colleagues in the 80s that tRNA and rRNA shared a common history [61]. It also supports recent findings of sequential and overlapping homologies of reconstructed tRNA with the PTC core of LSU rRNA [62].

In order to explore how the tRNA accretion process gave rise to functional rRNA, we traced the age of rRNA regions associated with relics of ancient tRNA building blocks (Figure 7). The ages of rRNA substructures were taken directly from ref. [11]. The oldest structural regions present in tRNA relics, for each relic, were highlighted as projections in the sequence of SSU and LSU rRNA and colored according to rRNA helix age (*nd*), from red (*nd* = 0; oldest) to blue (*nd* = 1; youngest). Relics were enriched in projections of old ribosomal regions (red, orange and yellow hues of the projections) that preceded the rise of the PTC and the “major transition” in ribosomal evolution (Figure 7). Note how projections of these old regions usually unify the many overlapping tRNA homologies, suggesting tRNA building blocks may have been at the beginning smaller and then slowly materialized into larger cloverleaf-like forms. In fact, and as we previously commented, tracing ribosomal protein history and tRNA interactions with domains in the phylogenetic timelines of ribosomal accretion revealed that a full-blown tRNA molecule was already interacting with the ribosome at the time of the major ribosomal transition [59]. Note the existence of substantial tRNA homology embedded in the PTC of LSU rRNA. Many tRNA homologies also showed substantial matching to other functional regions, including the central ratchet and hinges of SSU rRNA and the L1 and L7/12 stalks and the central protuberance (CP) of LSU rRNA that are involved in ribosomal dynamics. The most numerous overlapping tRNA matches coincided with old structures and involved tRNA with aminoacylation functions corresponding to the oldest Groups 1 and 2 that hold pre-transfer and post-transfer editing and trans-editing activities. In particular, tRNA relics encoding Ser and Leu were the most abundant (6 Leu and 5 Ser tRNAs, respectively) and matched the old central functional regions, supporting the ancestrality of editing specificities for the charging of these two amino acids (Figure 6b) and the proposal that they jumpstarted the “operational” genetic code [21]. This is expected since anticodon-binding domains responsible for major specificities of the standard genetic code appeared after the ribosomal transition (Figure 2).

**Figure 8:**
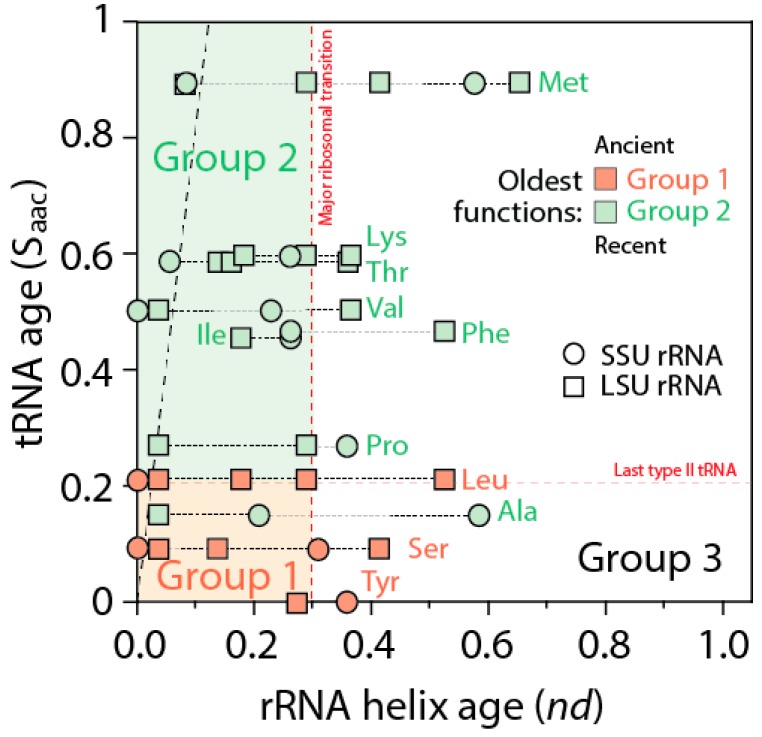
Coevolution of the most ancient rRNA substructures and tRNA holding the oldest aminoacylation functions (Groups 1 and 2) as these are pervasively coopted in rRNA. Ages were derived from phylogenetic constraint analysis of tRNA molecules [58] and from the timeline of ribosomal accretion inferred from the mappings of tRNA homologies to rRNA (see Figure 7). The more recent Group 3 aminoacylation functions are not plotted but are enriched in the white quadrant of the plot.

The ages of rRNA helices of the tRNA relics was also plotted against the age of tRNA isoacceptors derived from phylogenetic constraint analysis [58], which dissects the history of Groups 1 and 2 specificities in the timeline of tRNA accretion (Figure 8). The plot makes evident the ancestral nature of tRNA homologies and also shows the more recent tRNA recruitments. When considering homologies in the oldest rRNA segments, a coevolutionary pattern between the age of tRNA and tRNA building blocks of the ribosome appears evident (dashed line, Figure 8). The pattern suggests ribosomal construction by tRNA recruitment began very early and made use very quickly of the entire repertoire of tRNA isoacceptors derived from the editing specificities of their acceptor arms.

A close examination of Figure 7 allows postulating a succession of early cooption steps involving emerging Groups 1 and 2 isoacceptors into the growing ribosome. The primordial ribosomal ratchet of SSU and LSU moving parts (*nd* = 0–0.04) appeared to have been developed by cooptions of tRNA^Leu^, tRNA^Ser^, tRNA^Val^, tRNA^Pro^, and tRNA^Ala^ homologies. Similarly, the more derived SSU rRNA hinges (*nd* = 0.09–0.26) involved tRNA^Met^, tRNA^Ile^, tRNA^Phe^, and tRNA^Lys^ homologies. Finally, the rise of the ribosomal PTC (*nd* = 0.28–0.30) involved accretion of tRNA^Leu^, tRNA^Ser^, tRNA^Tyr^, tRNA^Pro^, tRNA^Met^, tRNA^Lys^ and tRNA^Thr^ homologies. The oldest Group 1 aminoacylation specificities appeared to have been remembered in the oldest structures of the ribosome, while the more derived Group 2 specificities are more abundant in later accretion steps.

## 10. Genomic Accretion of tRNA Building Blocks

When ancestral tRNA was translated in silico into proteins, its sequences showed homologies to elongation factors, aaRS enzymes, enzymes of nucleotide biosynthesis pathways and RNA polymerases [12]. Similar results were found in a separate study [63]. These remarkable results suggest both tRNA and tRNA ribosomal relics hold deep phylogenetic information indicating they both stored genetic information for ancient proteins and acted as ancient genomes. Modern biology provides important clues to this very primordial role of tRNA. Dispersed repetitive elements, especially those associated with tRNA, have the potential to spatially and functionally organize the genome by providing barriers to chromatin structure, DNA replication, and contributing to fragile sites prone to genomic rearrangements [64]. Synteny blocks in genomes, believed to be the result of chromosomal rearrangements, are often flanked by tRNA genes (e.g., [65]), suggesting an active role of tRNA encodings in genomic make up. Transposable elements often exhibit homologies to tRNA and have also active roles in the evolutionary restructuring of genomes [66]. The 3′-terminal ends of mRNAs in mitochondrial DNA are often immediately continuous to tRNA genes, which likely punctuate the polycistronic transcripts by endonucleolytic cleavage [67]. On this point, there is evidence that many aaRSs not only bind to their respective tRNA in order to catalyze esterification of the appropriate cognate amino acid, but also bind to homologous sequences on their own mRNA in order to carry out autogenous regulation of synthetase production. Examples include many of the Group 2 and 3 tRNAs, including the aaRS for tRNA^Thr^ [68,69,70,71,72,73,74,75,76,77,78,79], tRNA^Asp^ [80,81,82,83], tRNA^His^ [84], tRNA^Met^ [74], and tRNA^Phe^ [85]. Many other aaRSs (especially those in Groups 1 and 2) are also regulated by direct binding of the protein to genetic regulatory elements but not directly to their own mRNA [79,83]. Such autogenous control of synthetases provides additional evidence that tRNA may have played a central role not only in the origins of the ribosome, but also in the origins of the genome that encodes ribosome-related proteins. Finally, recent analysis of mimivirus transcripts shows tRNA genes are expressed as polyadenylated messengers and follow a stringent “hairpin rule”, which extends to the entire genome [86]. The ancestrality of giant viruses, and viruses in general [87], now suggests this oddity is an ancient (not derived) feature of the mimivirus genome. All of these properties support the crucial functional and structural role of genomic tRNA, boosting their ancient role as genomic building blocks.

## 11. Ribosomal Structure Supports rRNA and Genomic Evolutionary Growth from Primordial tRNA Pieces

The structural makeup of the ribosome provides information about its possible growth by covalent joining of primordial tRNA pieces [26]. Identification of putative insertions of “branch” helices onto preexisting coaxially stacked “trunk” helices in crystallographic models of the ribosome showed that not all insertions support the outward and gradual growth of ribosomal structures [88]. Seventeen putative insertions suggest either evolutionary events of inward growth or the existence of “structural grafting” of building blocks to build larger rRNA structures. The fact that these putative insertions flank regions with numerous tRNA homologies supports the idea that those building blocks were in fact primordial tRNA molecules (D. Caetano-Anollés, ms. in preparation).

## 12. Conclusions

**Figure 9:**
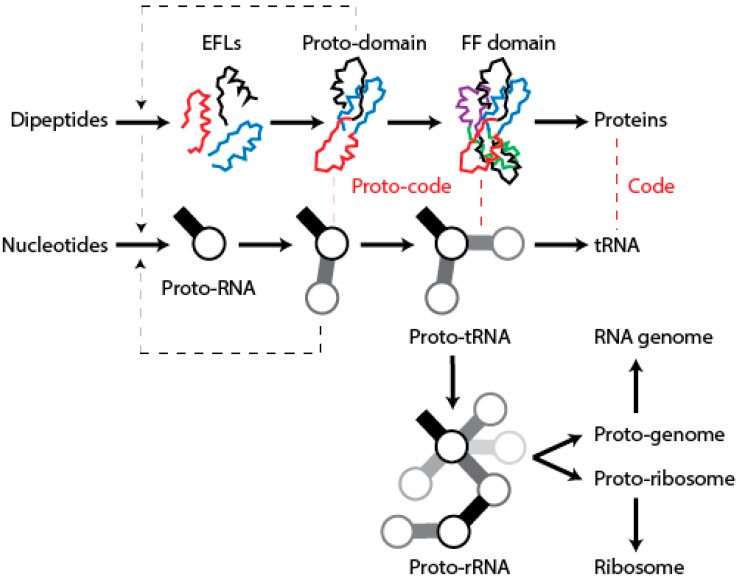
Model of evolutionary growth of macromolecules from component parts leading to translation machinery and genomes. Longer polypeptide molecules would have assembled from amino acids and dipeptides by statistically biased condensations. Some of these produced elementary functional loops (EFLs) capable of interacting with ligands and forming larger protein ensembles (EFLs with variant sequences and structures are illustrated with differently colored loop backbones). Similarly, proto-RNA molecules folding into small hairpins (stems are illustrated with solid bars and loops with open circles) assembled from nucleotides in EFL-delimited pockets and were later ligated to form larger RNA molecules serving as proto-genomes and proto-ribosomes. Interactions between RNA and emerging proteins establish primordial structural correspondences. This code of genetic memory is illustrated with red dashed lines. Black dashed arrows illustrate feed-forward catalytic activities.

We have postulated a phylogenomic data-driven evolutionary scenario describing the rise of translation and genetics [9]. It involves the lengthening of primordial cofactors into short RNA hairpins, which slowly gained compositional specificities and evolved into longer nucleic acid polymers protected by catalytic sites of the α/β/α-layered structures of archaic protein domains (summarized in Figure 9). Similarly, primordial protein domains likely assembled from smaller loop subunits, the EFLs [46]. This process of accretion of loop structures produced crucial domains, exemplified in the recently proposed emergence of class II aaRSs from three hairpin structures [89]. The initial protein-nucleic acid interactions resulted in “ternary complexes” of primordial aaRSs, translation factors, and tRNA, which aminoacylated tRNAs, ligated charged amino acids into dipeptides and longer polymers, and gradually gained specificities to ensure compositional memories would be preserved in proteins and interacting RNA [9]. These complexes were then “vectorially” transferred to other molecular contexts, which would give rise to more complex NRPS-like and ribosomal-like machinery. In particular, their interaction with newly formed OB-fold barrel structures produced an ancestor of the central ribosomal ratchet of SSU rRNA and its S12 and S17 ribosomal protein partners (the oldest of the ribosome) [11]. One important corollary of this scenario is that the specificities of the genetic code developed through stereochemical interactions between nucleic acid and protein molecules that were fully structured. In this regard and in line with the “self-referential model” for the origin of the genetic code [90], pockets in the α/β/α-layered structures of archaic synthetases were able to accommodate pairs of interacting RNA hairpins that were aminoacylated, catalyzing peptide bond formation. We believe the molecular environment of structural pockets resembled those of modern CDPSs, which foster the formation of tRNA-mediated dipeptidyl enzyme intermediates to produce a wide variety of dipeptides [91].

In the present study, phylogenetic tracings of ancient tRNA homologies in the ribosome reveal that cooption of emerging tRNA modules appears to be a protracted phenomenon responsible for both ribosomal structure and RNA “templating” memory. It is likely that the dynamics of cooption at RNA level responsible for rRNA and genomes, also brought with it interactions with emerging proteins domains. This resulted in a growing ribonucleoprotein ribosomal complex that was built gradually and from smaller pieces through protein-nucleic acid coevolution. It is also likely that numerous regulatory interactions involving tRNA mimicry at genomic level may have been established at this very early stage as a primordial and labile epigenetic (“paragenetic” *sensu* Alexander Brink) mechanism. These interactions evolved hand-in-hand with the emerging genetic machinery and ultimately gave rise to “*field(s) of possibilities*”, the genes of genomes [92].

## Abbreviations

The following abbreviations are used in this manuscript:

aaACPL	amino acid-[acyl carrier protein]-ligase
aaRS	aminoacyl-tRNA synthetase
CDPS	cyclodipeptide synthase
EFL	Elementary functional loop
FF	Fold family
FSF	Fold superfamily
NRPS	non-ribosomal peptide synthetase
PTC	peptidyl transferase center
SCOP	Structural classification of proteins
